# VITOM 4K 3D Exoscope: A Preliminary Experience in Thyroid Surgery

**DOI:** 10.7759/cureus.12694

**Published:** 2021-01-14

**Authors:** Peter Kullar, Ravina Tanna, Munira Ally, Ananth Vijendren, George Mochloulis

**Affiliations:** 1 Otolaryngology, East and North Hertfordshire NHS Trust, Stevenage, GBR

**Keywords:** vitom, exoscope, thyroid surgery, novel, technology, ent, imaging

## Abstract

Since its introduction in 2008, the Karl Storz 4K 3D VITOM® exoscope (Karl Storz SE & Co. KG, Tuttlingen, Germany) has been successfully used in various surgical disciplines. This paper describes our department’s experience using this technology and its use in the first total thyroidectomy case. The set up of the 3D VITOM exoscope in the operating theatre allows for a user-friendly approach to thyroid surgery with the exoscope placed out of the line of sight of the surgeon with a monitor placed directly ahead. The surgeon has a control panel within reach, which allows for adjustments to image magnification and focus. The use of the 3D VITOM exoscope has the potential to confer significant improvements in patient outcomes by promoting efficient and safer surgery through superior operative visualisation.

## Introduction

Surgical resection of one or both thyroid lobes is recommended for both the treatment of benign conditions, including thyrotoxicosis and goitre, and in the treatment of suspicious nodules or thyroid malignancy. Although a commonly performed procedure, with approximately 500 thyroidectomies undertaken in the UK each month, it is associated with significant post-operative complications [1]. These include haematoma, wound infection, transient and permanent hypocalcaemia and recurrent laryngeal nerve injury [2]. The rates of these complications are directly related to the type and extent of the disease and the experience level of the surgeon [3]. Unilateral recurrent laryngeal nerve (RLN) palsy results in significant quality of life changes, whereas bilateral RLN palsy may lead to life-threatening airway obstruction. The rate of permanent RLN palsy has been reported to range between 0.3% to 10% [4]. Post-operative hypocalcaemia has been reported to occur in 23.6% of patients undergoing total thyroidectomy [1]. As well as causing significant morbidity, hypocalcaemia affects the length of patient stay and necessitates the use of repeated blood tests to guide management [5]. Therefore, there is a pressing need for surgical technologies that minimise the risk of surgical complications [6].

The operating microscope and endoscope are well established in modern surgical practice and have found particular application in otolaryngology [7]. Recently, the Karl Storz 4K 3D VITOM® (video-assisted telescope operating monitor) (Karl Storz SE & Co. KG, Tuttlingen, Germany) exoscope has been introduced as an adjunct or alternative to these traditional optical devices. It has been reported that the 3D exoscope system offers advantages to the operating microscope in other surgical disciplines, particularly neurosurgery and ophthalmology, however, we present the first case of total thyroidectomy completely undertaken using the 4K 3D VITOM exoscope [8].

## Case presentation

Method

This operation was performed on a 47-year-old female with a three-year history of hyperthyroidism resistant to medical management. Ultrasound imaging demonstrated an 8.2 cm multinodular goitre. The patient underwent a total thyroidectomy using the 4K 3D VITOM for the entire procedure.

The 4K 3D VITOM consists of a 4K endoscope and a 300W xenon fibreoptic light source. The VITOM is mounted external to the body cavity and is positioned using a pneumatic arm. The camera provides 8-30x magnification and a depth of field between 7-44 mm, allowing a working distance of 20-50 mm. The system projects to a 3D 55-inch monitor, with a maximum screen resolution of 1920 × 1080 in 16:9 image format, requiring the surgical team to wear 3D passive-polarised glasses. These have a different filtering system for each eye, meaning each eye only sees the image intended for it. They see the left and right image at the same time onto the area through polarised filters. These filters are oriented at right angles to each other. Each eye perceives a different image resulting in the 3D effect. The monitor is positioned to permit line-of-sight surgery, removing the necessity to physically interact with any magnifying device. The system is controlled by a slave unit comprising a wheel and joystick, which can be used to manipulate the micro-position, focus, and zoom of the camera.

**Figure 1:**
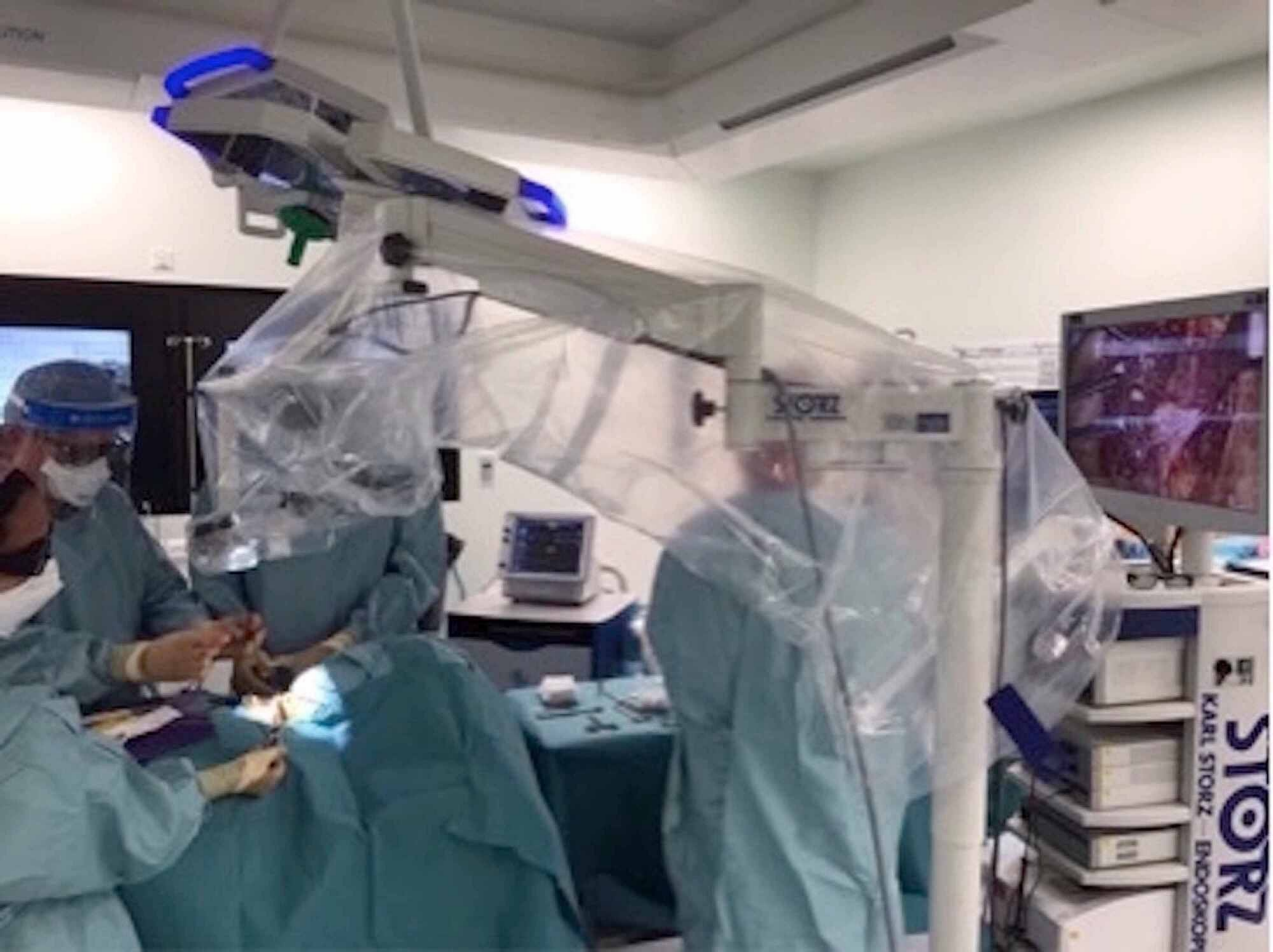
Position of the VITOM® 3D exoscope system in the operating theatre

**Figure 2:**
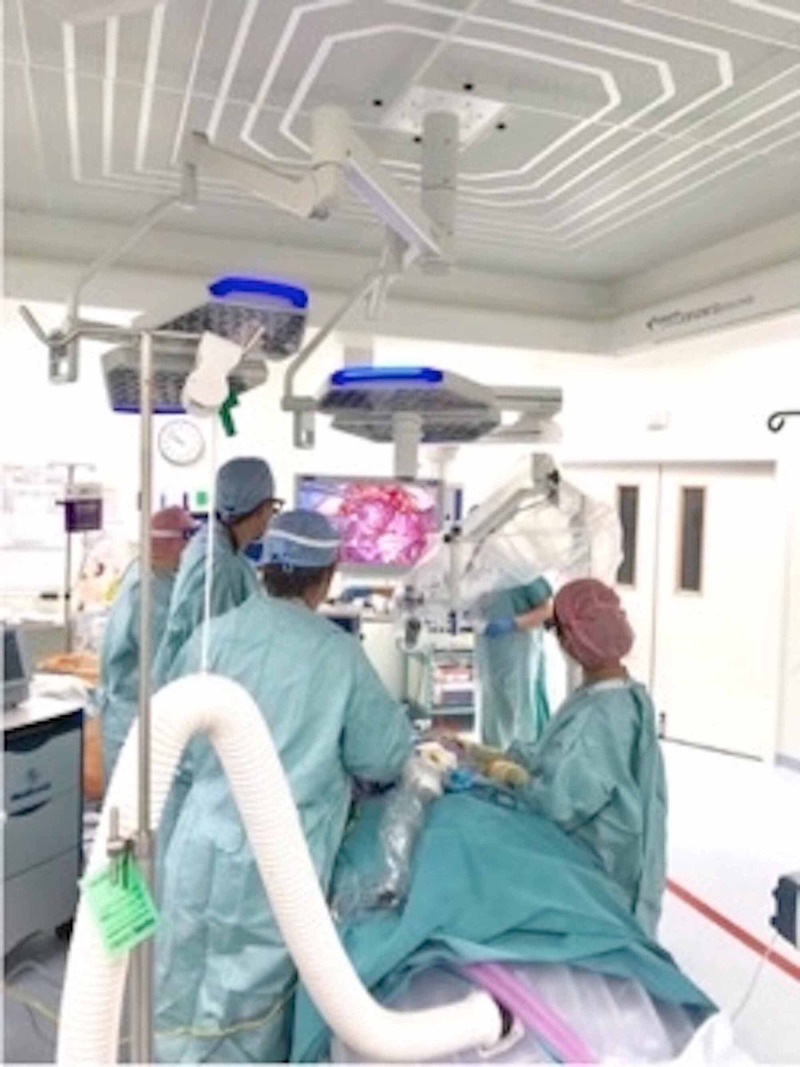
Position of the surgeons in the operating theatre

The procedure was carried out with the patient in a supine position on a head ring and shoulder roll. The exoscope, within a sterile drape, was placed 50 cm from the patient to the right of the surgeon and outside the line of sight. It projected to a 3D monitor, placed 2.5 m from the surgeon, positioned at eye level directly opposite the operating surgeon. The control panel was placed to the left of the surgeon within reach, allowing adjustment of focus and fine position of the field of view. The scrub nurse was positioned at the head and the anaesthetist at the foot of the bed. (Figure 1, 2) 

Surgical technique

The patient was intubated with a neural integrity monitor or NIM® (Medtronic, Minneapolis, USA) electromyogram endotracheal tube. Following a 5 cm transverse skin crease incision, the dissection was continued to the infrahyoid strap muscles. Continuous nerve monitoring was used by fitting an Automatic Periodic Stimulation (APS™)probe (Medtronic, Minneapolis, USA) to the ipsilateral vagus nerve in the carotid sheath [9]. The upper pole of the thyroid is first dissected using the LigaSure™ (Medtronic, Minneapolis, USA) followed by the lower pole and the position of the RLN is confirmed using a handheld probe. After the removal of both thyroid lobes, the APS probe was removed, the strap muscles were approximated, and the wound closed in layers. The procedure was recorded on the hard drive of the VITOM system. The procedure lasted 75 minutes. There were no immediate complications and the patient was discharged the next day on a standard post-operative regime of calcium and thyroxine supplementation. The histological diagnosis confirmed benign multinodular goitre. Follow up at four weeks revealed a well-healed incision, normal vocal cord movements, and a normal calcium level.

## Discussion

The 4K 3D VITOM exoscope represents a significant advance in intraoperative imaging. This surgical imaging technology enables a heads-up display similar to an endoscope, but it is positioned external to the operating field in a similar manner to an operating microscope. Currently, four exoscopic systems are available: the VITOM (Karl Storz), KINEVO® (Carl Zeiss AG, Oberkochen, Germany), Modus V™ (Synaptive Medical, Toronto, Canada) and ORBEYE® (Olympus Corporation, Tokyo, Japan).

Since its introduction in 2008, the VITOM has been employed in diverse surgical fields, including neurosurgery, paediatrics, gynaecology, urological, and ear nose throat (ENT) surgery [10-12]. To our knowledge, we report the first described case of a total thyroidectomy performed using the 4K 3D VITOM. Our experience demonstrates the feasibility and advantages of using the system for thyroid surgery. The main features that differentiate the VITOM 3D from traditional surgical techniques include ergonomics, surgical visualization, versatility, and its use as a training tool.

Ergonomics

Occupational musculoskeletal injuries are prevalent amongst surgeons and have the potential to shorten operative careers [13]. Maintaining a static body position with extended periods of time in neck flexion is an important contributing factor in the 72% of UK ENT surgeons who experience neck and/or back pain [14]. Thyroidectomy requires an operative position with prolonged neck flexion of approximately 30 degrees [13]. The VITOM system provides a considerable advantage in allowing a neutral cervical spine position and natural spinal posture. Some surgeons may consider wearing 3D glasses for a prolonged period uncomfortable, and some initially experience a sense of dysequilibrium, however, we found that the majority quickly adapt. The wearing of 3D glasses both enables the viewing of magnified 3D real-time images and protects the eyes from aerosols. This is of particular importance during the current COVID-19 pandemic [15].

Surgical visualisation

The VITOM system allows a depth of field between 3.5 cm and 10 cm and a magnification of 12-30x, which compares favourably with the ENT operative microscope. The 3D VITOM permits full stereopsis at magnification and the addition of the colour filters (Clara/Chroma/Spectra) highlight the interface between tissue types including blood vessels and nerves. This aids the identification of critical structures including the RLN and vessels allowing precise use of electrocautery for hemostasis. It is our expectation this will make a meaningful impact on post-operative complication rates. Additionally, surgeons are easily able to shift between the VITOM image and macroscopic visualisation without removing the glasses. The ability to use both techniques is particularly important as the surgeon adapts to using the device.

Versatility

The VITOM is highly manoeuvrable and can be positioned outside the line of sight of the surgeon and assistants whilst allowing full visualisation of the surgical field. It is important to note that in order to obtain an in-focus view of the surgical field, the screen must be oriented at the surgeon’s eye level, otherwise, a marked image degradation is noted. This may cause problems when individuals within the surgical team are of different heights. Although currently repositioning of the VITOM is required during operating, the introduction of the robotic ARTip™ Cruise (Karl Storz SE & Co. KG) robotic system will considerably reduce operative interruption. This is broadly speaking a robotic holding arm controlled by the standard VITOM 3D joystick and a foot-pedal [11]. We feel the VITOM system gives the benefits of visualisation achieved with robotic surgery without the loss of haptic feedback.

Teaching

We found the VITOM provided a significant advantage for training and education. The 3D view of the primary surgeon can be shared with the whole team, both increasing engagement and facilitating surgical training [16]. In particular, both scrubbed and assistant nurses can directly observe the surgical field and anticipate the surgeon’s choice of instrument. This guarantees a real immersive surgical experience for all participants. Additionally, procedures can be recorded by the system, meaning, telesurgery and telementoring can be facilitated. This is particularly pertinent during the COVID-19 pandemic, where surgical learning is being considerably adapted to involve the use of online resources.

## Conclusions

The 4K VITOM 3D offers distinct advantages over traditional surgical techniques. The information gained from our experience in successfully performing the first total thyroidectomy using the exoscopic system will enable us to generate hypotheses testable in future studies. Specifically, we envisage the system may be used to promote more efficient and safer surgery and thereby improve patient outcomes.
